# To identify normovolemia in humans: The stroke volume response to passive leg raising vs. head‐down tilt

**DOI:** 10.14814/phy2.15216

**Published:** 2022-07-19

**Authors:** Casper Sejersen, Till Christiansen, Niels H. Secher

**Affiliations:** ^1^ Department of Anaesthesia Institute for Clinical Medicine University of Copenhagen Copenhagen Denmark

**Keywords:** cardiac output, cardiovascular regulation, central blood volume, stroke volume, thoracic electrical admittance, Trendelenburg's position

## Abstract

Volume responsiveness can be evaluated by tilting maneuvers such as head‐down tilt (HDT) and passive leg raising (PLR), but the two procedures use different references (HDT the supine position; PLR the semi‐recumbent position). We tested whether the two procedures identify “normovolemia” by evaluating the stroke volume (SV) and cardiac output (CO) responses and whether the peripheral perfusion index (PPI) derived from pulse oximetry provides similar information. In randomized order, 10 healthy men were exposed to both HDT and PLR, and evaluations were made also when the subjects fasted. Central cardiovascular variables were derived by pulse contour analysis and changes in central blood volume assessed by thoracic electrical admittance (TEA). During HDT, SV remained stable (fasted 110 ± 16 vs. 109 ± 16 ml; control 113 ± 16 vs. 111 ± 16 ml, *p* > 0.05) with no change in CO, TEA, PPI, or SV variation (SVV). In contrast during PLR, SV increased (fasted 108 ± 17 vs. 117 ± 17 ml; control 108 ± 18 vs. 117 ± 18 ml, *p* < 0.05) followed by an increase in TEA (*p* < 0.05) and CO increased when subjects fasted (6.7 ± 1.5 vs. 7.1 ± 1.5, *p* = 0.007) with no change in PPI or SVV. In conclusion, SV has a maximal value for rest in supine men, while PLR restores SV as CBV is reduced in a semi‐recumbent position and the procedure thereby makes healthy volunteers seem fluid responsive.

## Graphical abstract

Volume responsiveness can be evaluated by tilting manoeuvres such as head‐down tilt (HDT) and passive leg raising (PLR), but the two procedures use different references (HDT the supine position; PLR the semi‐recumbent position). Stroke volume has a maximal value for rest in supine men, while PLR restores SV as central blood volume is reduced in a semi‐recumbent position and the procedure thereby makes healthy volunteers seem fluid responsive.

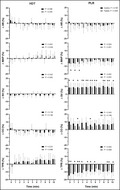

## 1 INTRODUCTION

Normovolemia has been defined as the volume that provides the heart with a central blood volume (CBV) that does not limit cardiac pumping capacity and manifests during supine rest for young healthy volunteers (Harms et al., 2003). Conversely, hypovolemia may be considered as a reduction in preload to the heart, i.e., stroke volume (SV) and cardiac output (CO) become dependent on CBV. A measure of early reduction in CBV is SV (Cooke et al., 2004) and an increase in SV in response to administration of volume implies that the heart is preload‐responsive (Pinsky, 2002). With that perspective, volume treatment could be provided to ensure a resting SV (and thus CO) that is not limited by preload to the heart.

Goal‐directed fluid therapy (GDT) aims to optimize oxygen delivery, often by evaluating the SV response to administration of, e.g., 200 ml colloid considering that a 10% increase indicates a need for administration of fluid (Bundgaard‐Nielsen et al., 2010b). Alternatively, volume responsiveness may be assessed by exposing the patient to head‐down tilt (HDT; Trendelenburg's position), again supposing that an increase in SV by 10% indicates a need for volume administration, and also passive leg raising (PLR) is applied to evaluate whether patients are in need of expanding the circulating blood volume (Thiel et al., 2009).

The PLR has been proposed as an attractive way to predict fluid responsiveness with diagnostic accuracy in meta‐analyses (Cavallaro et al., 2010; Cherpanath et al., 2016) and is advocated by the European Society of Intensive Care Medicine (Cecconi et al., 2014) to evaluate whether a patient is in need of expanding the circulating blood volume, the Surviving Sepsis Campaign (Rhodes et al., 2017) and National institute for Health and Care Excellence (NICE, 2013). As stated by NICE, PLR is best undertaken with the patient semi‐recumbent and then tilting the bed 45° to rise the legs promoted as “five rules and not a drop of fluid” by Monnet and Teboul (2015) referring to the positional sequence. However, the HDT and PLR procedures use different references: for HDT the supine position; for PLR as mentioned a semi‐recumbent position (the upper body raised 45° with the legs in a horizontal position). For normal young people, there is no increase in SV when moving from a supine position to HDT although central venous and pulmonary artery pressures increase (Harms et al., 1999, 2003), suggesting that the heart is working on the upper horizontal part of the Starling curve when humans are supine. Thus, in supine humans, SV may provide a means for evaluating how much fluid should supplement the blood volume, if any. In contrast, we suspected that CBV is reduced in the semi‐recumbent position used in the PLR procedure, and raising the legs would increase SV as a consequence of restoring CBV.

In this randomized study, healthy men were exposed to both HDT and PLR. We hypothesized that an increase in SV during PLR is by an increase in CBV, while there would be no SV response in consequence of HDT. For the results to be relevant to patients who are to go through surgery, evaluations were made both with the subjects fasting and after a normal breakfast (control), and compared to the studies by Harms et al. (1999, 2003), subjects were chosen to represent a wide age span. In addition to the determination of central cardiovascular variables, changes in CBV were assessed by thoracic electrical admittance (TEA) (Cai et al., 2000b; Matzen et al., 1991). Furthermore, heart rate variability (HRV) was determined as it is often used to indicate autonomic control of the heart (Xhyheri et al., 2012). Oxygen saturation (SpO_2_) measured non‐invasively is applied in clinical practice and thus readily available to provide an albeit indirect measure of changes in SV from the peripheral perfusion index (PPI) by photo‐plethysmography (Goldman et al., 2000) and therefore also recorded.

## 2 MATERIALS AND METHODS

### 2.1 Ethical approval

This study was approved by the Central Denmark Regional Committees on Health Research Ethics (reg.no. 1‐10‐72‐212‐17) in accordance with the Declaration of Helsinki except for registry in a database. After oral and written information, all participants gave written consent prior to participation.

### 2.2 Participants

Ten healthy male volunteers participated in the study [median and range: age 39 (21–74) years, height 177 (172–194) cm, weight 80 (69–136) kg, and thus a BMI of 25 (22–43) kg/m^2^]. The subjects were not taking any prescribed medication and were free of cardiovascular, metabolic, or neurological diseases. Also, the subjects refrained from caffeinated beverages for 12 h and consumed either a normal breakfast including fluid (e.g., milk products and juice) ad libitum or fasted for at least 6 h prior to visiting the laboratory as typically instructed before elective surgery. The sample size was calculated a priori based on SV during PLR (unpublished data) estimating that 10 volunteers would provide sufficient power (80%) to detect significance based on an α = 0.05.

### 2.3 Experimental protocol

**FIGURE 1:**
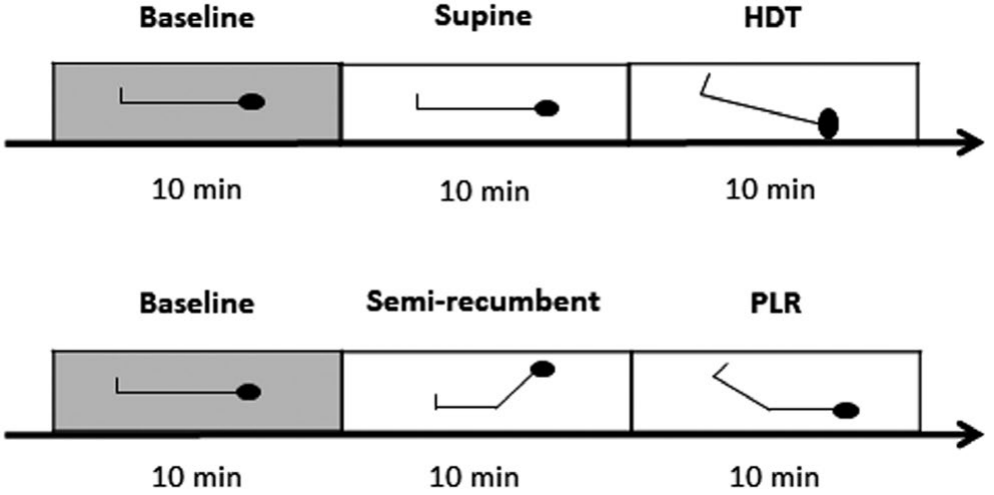
Representation of the two maneuvers. Both maneuvers were carried out during each intervention (fasted and control) and the order was randomized. The maneuvers were following each other, but here separated. HDT, head‐down tilt; PLR, passive leg raising

The participants visited the laboratory on two occasions separated by at least 24 h. The protocol included the two tilting maneuvers: HDT and PLR during each visit with the order randomized. Also, the participants were randomized to be either fasted or controlled. Both protocols included a 10 min supine period to establish baseline values. During HDT, the participants were supine on a motor‐driven tilt‐table for another 10 min and then exposed to 20⁰ HDT for 10 min with support for the shoulders (Figure 1). During PLR, the participants were tilted from a supine control position to a semi‐recumbent position with the back 45° upright and the legs horizontal for 10 min. Thereafter, the legs were elevated 45⁰ with the upper body in a horizontal position (PLR) for another 10 min.

### 2.4 Measures

Arterial pressure and heart rate (HR) were recorded using photoplethysmography (Nexfin, BMEYE B.V., Amsterdam, the Netherlands) with the finger cuff at the third middle phalanx of the right hand and a heart reference sensor mounted to report values at the level of the heart (Bogert & Van Lieshout, 2005). A pulse contour method (Nexfin CO‐trek, BMEYE B.V.) adapted for age, gender, height, and weight (Bogert et al., 2010; Truijen et al., 2018) provided left ventricular SV and CO as SV times HR. Non‐invasive determination of cardiovascular variables by Nexfin have been validated, e.g., during cardiothoracic surgery (Truijen et al., 2018) and HDT and head‐up tilt (Harms et al., 1999). Variation in SV was calculated as $\mathrm{SVV}=\;\frac{{\mathrm{SV}}_{\max}‐\;{\mathrm{SV}}_{\min}}{{\mathrm{SV}}_{\mathrm{mean}}}$, where SV_max_ is the maximal value, SV_min_ the smallest, and SV_mean_ the mean of 5 s time segments and a mean of each segment calculated. The SVV is considered a dynamic measure of preload in heart‐lung interaction sensitive to preload: changes in preload induced by ventilation impact SVV. Total peripheral resistance (TPR) was $\frac{\mathrm{MAP}}{\mathrm{CO}}$. Another plethysmographic module installed in the Nexfin apparatus (Masimo, Irvine, California, USA) derived SpO_2_ and PPI from the pulse oximetry signal obtained on the right index finger with PPI reflecting the ratio between the pulsatile and non‐pulsatile component of the arterial waveform (Goldman et al., 2000). The PPI was calculated as $\frac{\mathrm{AC}}{\mathrm{DC}}100\%$, where AC represents the variable amount of infrared light absorbed by pulsating arterial inflow and DC the constant absorption of infrared light due to skin and other tissues. Thus, PPI assesses peripheral vasomotor tone with changes reflecting the pulsatile component of the signal and the ratio changes by manipulation of CBV and thereby SV during, e.g., lower body negative pressure (Van Genderen et al., 2013).

The participants were instrumented with electrocardiogram (ECG) electrodes (Q‐10‐25, Ambu^®^, Denmark) for the calculation of HRV. Sampled at 1000 Hz with a PowerLab data‐acquisition device (ADinstruments Ltd., Oxford, UK), a high (HF, 0.15–0.4 Hz) and low (LF, 0.04–0.15 Hz) frequency power of the RR interval in the ECG were extracted to derive indices of parasympathetic and sympathetic activity (Tarvainen et al., 2014, 2020).

Also, a pair of electrodes (N‐00‐25, Ambu^®^) were placed on the right sternocleidomastoid muscle and another pair high in the left mid‐axillary line, each pair separated by ~5 cm to estimate changes in CBV by TEA. Evaluation of TEA was based on an excitation current of 200 µA at 1.5 and 100 kHz (C‐Guard, Danmeter, Denmark) with the outer electrodes providing the current and the inner pair determining TEA. The low‐frequency current does not penetrate the cell lipid membrane readily and thus reflects the extracellular volume. Conversely, the high‐frequency current correlates to total (regional) body water as it penetrates the cell membrane (Cai et al., 2000b). Thus, changes in the difference between TEA (1/impedance, IDX) at a low‐ and a high‐frequency current is taken to reflect changes in intracellular volume and therefore presumably in the regional red cell volume (Cai et al., 2000a,b). The sampling rate was 2 Hz and values integrated over 15 s to minimize respiratory influence. To time‐align variables, markers were placed simultaneously.

### 2.5 Statistical analysis

Descriptive data are presented as mean ± SD over the last 30 s of rest and every minute for each intervention besides TEA for every 5th min. The ECG was exported and bandpass filtered (Kubios, Kuopio, Finland) with data extracted into 5 min recordings. Raw data from the Nexfin apparatus were inspected and beats without apparent artifacts selected. A Shapiro‐Wilk test evaluated data distribution. A one‐way ANOVA with repeated measures was used to identify differences and identified with the Bonferroni post hoc test. Statistical significance was set at *p* < 0.05 using SPSS statistics 26 (IMB, Armonk, New York, USA).

## 3 RESULTS

Two participants did not complete both interventions as one withdrew from the study and one had other engagements. Thus, data are presented for nine volunteers when fasted and nine during control with consistent SV responses among subjects.

### 3.1 HDT

#### 3.1.1 Control

**FIGURE 2:**
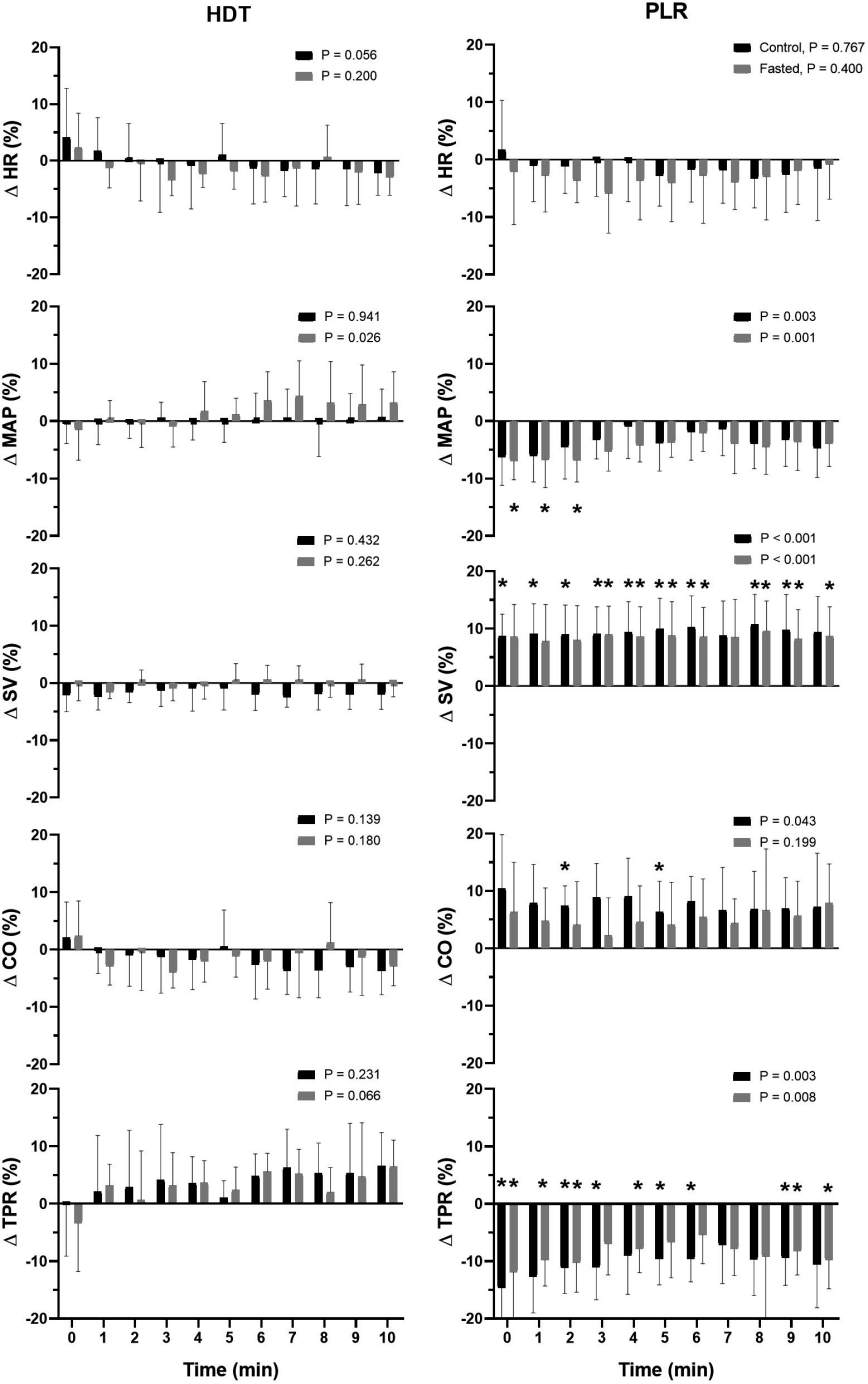
Change from reference point in heart rate (HR), mean arterial pressure (MAP), stroke volume (SV), cardiac output (CO), and total peripheral resistance (TPR) during 10 min head‐down tilt (HDT) and passive leg raising (PLR). Data are mean ± SD. *p*‐value represents evaluation by ANOVA. *Compared to reference point, *p* < 0.05

**FIGURE 3:**
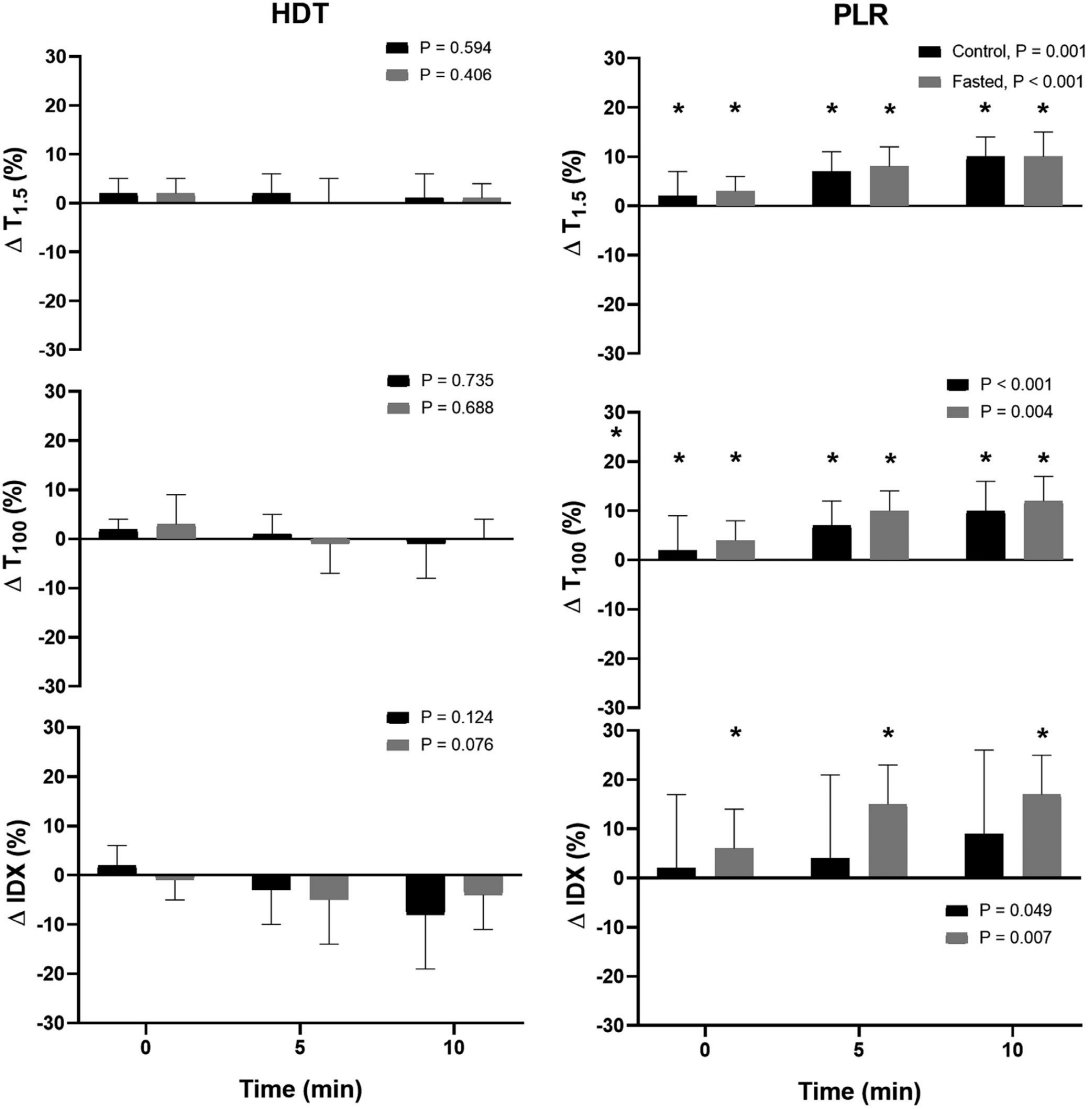
Change in thoracic electrical admittance at 1.5 (T_1.5_) and 100 kHz (T_100_) and the index value (IDX) during 10 min head‐down tilt (HDT) and passive leg raising (PLR). Data are mean ± SD. *p*‐value represents evaluation by ANOVA. *Compared to reference point, *p* < 0.05

**FIGURE 4:**
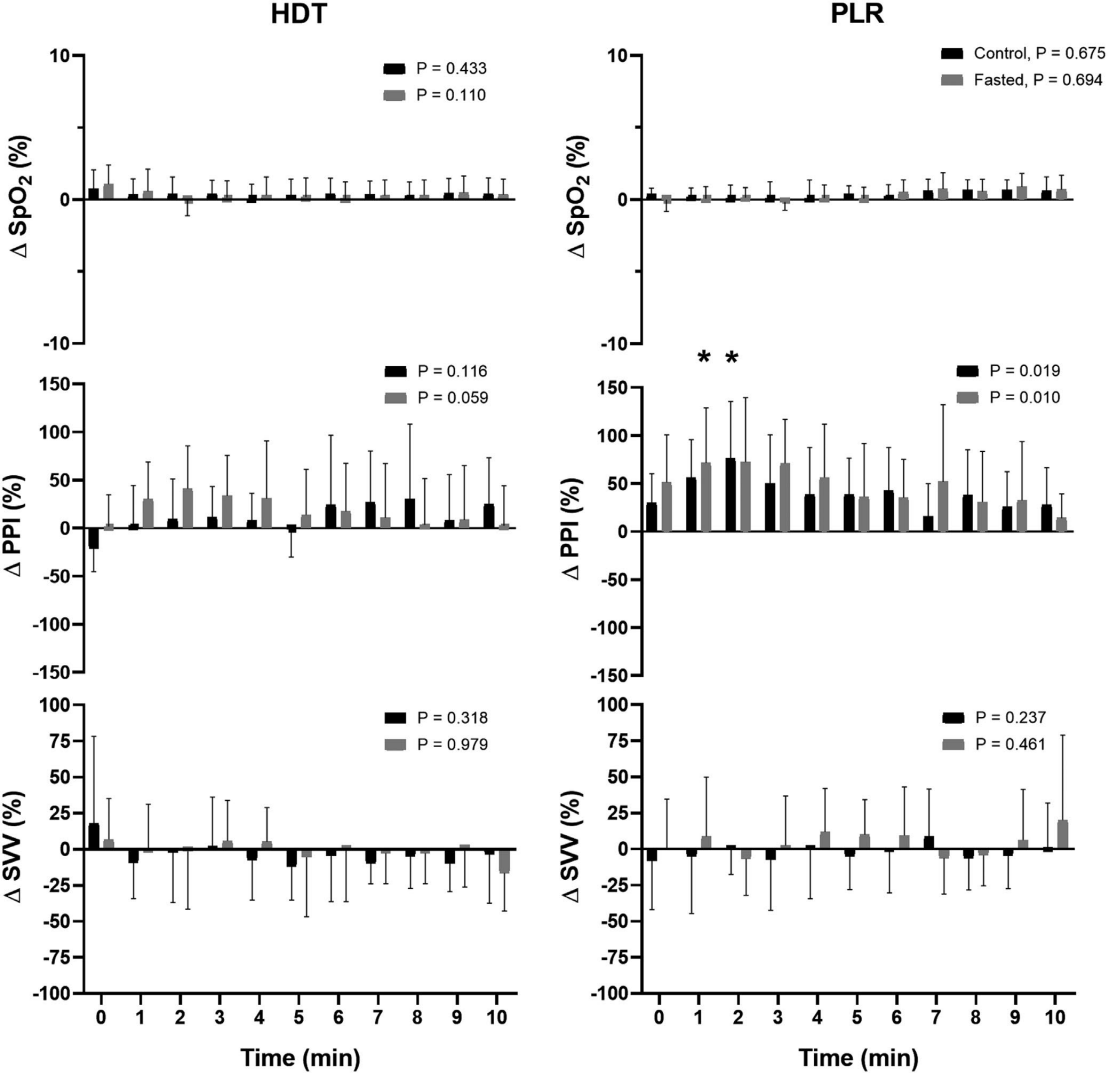
Change in peripheral perfusion index (PPI) and stroke volume variation (SVV) during 10 min head‐down tilt (HDT) and passive leg raising (PLR). Circles are individual data and boxes are mean ± SD. *p*‐value represents evaluation by ANOVA. *Compared to reference point, *p* < 0.05

There were no differences in any variable between baseline and supine rest. Also, when exposed to HDT, the cardiovascular and TEA variables remained similar (Table 1) and were unaffected throughout the 10 min period besides a tendency toward an increase in HR at the beginning of the maneuver by 4 ± 9% (*p* = 0.056; Figures 2, 3, 4).

**TABLE 1:** Hemodynamic variables during the last minute of each intervention, thoracic electrical admittance, and heart rate variability during the last 5 min of supine rest, head‐down tilt (HDT), semi‐recumbent, and passive leg raising (PLR)

Tilt angle	Supine		HDT		Baseline		Semi‐recumbent		PLR	
	Fasted	Control	Fasted	Control	Fasted	Control	Fasted	Control	Fasted	Control
MAP (mmHg)	83 ± 8	86 ± 10	85 ± 8	86 ± 8	87 ± 8	85 ± 12	91 ± 8*	90 ± 11*	88 ± 7	87 ± 11
HR (beats min^−1^)	62 ± 8	62 ± 11	60 ± 8	62 ± 10	64 ± 7	62 ± 6	62 ± 6	63 ± 7	61 ± 9	62 ± 9
SV (ml)	110 ± 16	113 ± 16	109 ± 16	111 ± 16	110 ± 18	112 ± 16	108 ± 17	108 ± 18	117 ± 16*,**	117 ± 18*,**
CO (l min^−1^)	7 ± 2	7 ± 2	7 ± 1	7 ± 2	7 ± 2	7 ± 1	7 ± 1	7 ± 1	7 ± 2**	7 ± 1
TPR (mmHg l min^−1^)	13 ± 3	13 ± 4	14 ± 3**	13 ± 4	14 ± 4	13 ± 3	14 ± 4	14 ± 3	13 ± 4**	13 ± 3**
SVV (%)	10 ± 4	9 ± 4	8 ± 4	8 ± 4	9 ± 5	9 ± 5	11 ± 5	11 ± 4	9 ± 4	9 ± 3
PPI (%)	6 ± 2	6 ± 3	6 ± 1	6 ± 3	6 ± 4	5 ± 2	3 ± 2	3 ± 1	4 ± 1	4 ± 2
SpO_2_ (%)	97 ± 2	97 ± 2	97 ± 1	98 ± 2	97 ± 2	97 ± 2	97 ± 2	97 ± 2	97 ± 1	98 ± 2
T_1.5_ (S)	172 ± 25	180 ± 31	173 ± 27	184 ± 34	175 ± 30	181 ± 33	158 ± 27*	163 ± 28*	174 ± 42**	178 ± 33**
T_100_ (S)	235 ± 41	238 ± 47	234 ± 42	239 ± 52	238 ± 48	239 ± 50	211 ± 41*	213 ± 42*	236 ± 42**	234 ± 52**
IDX (S)	64 ± 18	61 ± 17	61 ± 16	57 ± 19	63 ± 19	58 ± 20	53 ± 16*	52 ± 17	63 ± 16**	59 ± 21
HF (n.u.)	42 ± 15	43 ± 20	41 ± 17	40 ± 23	35 ± 14	44 ± 18	37 ± 16	37 ± 26	33 ± 13	40 ± 19
LF (n.u.)	58 ± 15	57 ± 20	59 ± 17	60 ± 23	65 ± 14	56 ± 18	63 ± 16	63 ± 26	67 ± 13	60 ± 19
LF/HF	2 ± 1	2 ± 1	2 ± 2	2 ± 2	2 ± 1	1 ± 1	3 ± 3	3 ± 3	3 ± 2	2 ± 2
PNS index	1 ± 2	1 ± 2	1 ± 2	1 ± 2	1 ± 2	1 ± 2	1 ± 1	1 ± 2	1 ± 2	1 ± 2
SNS index	0 ± 1	0 ± 1	0 ± 2	0 ± 1	0 ± 1	0 ± 1	0 ± 1	0 ± 1	−1 ± 1	0 ± 1

Note

*p < 0.05 from baseline; **p < 0.05 from prior tilt angle.

Abbreviations: CO, cardiac output; HF, high frequency heart rate variability; HR, heart rate; IDX, index value; LF, low frequency heart rate variability; LF/HF, low and high frequency ratio; MAP, mean arterial pressure; PNS index, parasympathetic index; PPI, peripheral perfusion index; SNS index, sympathetic index; SpO_2_, oxygen saturation; SV, stroke volume; SVV, stroke volume variation; T_1.5_, thoracic electrical admittance at 1.5 kHz; T_100_, thoracic electrical admittance at 100 kHz; TPR, total peripheral resistance.

#### 3.1.2 Fasted

There were no differences between control and fasted subjects. Also, there were no differences between baseline and supine rest variables and the cardiovascular and TEA variables remained unaffected by HDT except for an increase in TPR (to 13.8 ± 3.4 mmHg l min^−1^, *p* = 0.020) (Figures 2, 3, 4).

### 3.2 PLR

#### 3.2.1 Control

When tilted from supine rest to the semi‐recumbent position, HR remained similar while MAP increased (to 90 ± 11 mmHg, *p* = 0.012) (Table 1). No change occurred in SV, CO, or TPR even though both the reflection of thoracic extracellular volume (T_1.5_; to 163 ± 28, *p* = 0.002) and total body water (T_100_; to 213 ± 42 S, *p* = 0.013) decreased with no change in the estimated intracellular volume (IDX). The PPI, SpO2, SVV, and HRV remained unaffected by tilting to the semi‐recumbent position.

The PLR did not affect HR or MAP, and these remained unaffected for the 10 min procedure (Table 1 and Figure 2). The SV increased (to 117 ± 18 ml, *p* = 0.025) and the about 10% increase was withheld throughout the procedure providing for an about 8% increase in CO although only significant at min 2 (*p* = 0.04) while TPR decreased (to 12.8 ± 1.4 mmHg l min^−1^, *p* = 0.009). The increase in SV was followed by an increase in both thoracic extracellular volume (T_1.5_; to 178 ± 33 S, *p* = 0.005) and total body water (T_100_; to 234 ± 52 S, *p* = 0.019) but not in intracellular volume (IDX; *p* = 0.159). These changes (T_1.5_; by 10 ± 4% and T_100_; by 10 ± 6%) manifested during the last 5 min of PLR (Figure 3). Overall, the PPI did not change but when viewed over the 10 min, PPI increased within the first minutes and then normalized while SpO_2_, SVV, and HRV remained unaffected by PLR during the whole 10 min (Figure 4).

#### 3.2.2 Fasted

There were no differences between control and fasted. Thus, similar to control changing from supine rest to the semi‐recumbent position, HR remained unchanged while MAP increased (to 91 ± 8 mmHg, *p* = 0.014). No change occurred in SV or TPR but there was a tendency toward a decrease in CO (*p* = 0.056). The reflection of thoracic extracellular volume (T_1.5_; to 158 ± 27, *p* < 0.001), total body water (T_100_; to 211 ± 41 S, *p* < 0.001), and also estimated intracellular volume (IDX; to 53 ± 16, *p* = 0.002) decreased. The PPI, SpO2, SVV, and HRV remained unaffected (Table 1).

Similar to control, the PLR did not affect HR or MAP (Table 1), although MAP decreased during the first 2 min whereas HR remained unaffected (Figure 2). The SV increased (to 117 ± 16 ml, *p* < 0.005) also by about 10% with the increase being withheld during the 10 min procedure (Figure 2). Thus CO increased (to 7.1 ± 1.7 l min^−1^, *p* = 0.007) and TPR decreased (to 13.4 ± 3.9 mmHg l min^−1^, *p* = 0.003) with the decrease occurring from the beginning of the maneuver and withheld throughout the 10 min. Similar to control, the reflection of thoracic extracellular volume (T_1.5_), total body water (T_100_), but also intracellular volume (IDX; to 63 ± 16 S, *p* < 0.001) became larger throughout PLR (Figure 3). Similar to control, PPI did not change overall but increased during the first minutes, while SpO_2_, SVV, and HRV remained unaffected by PLR (Figure 4).

## 4 DISCUSSION

This study addressed the SV and CO responses to two procedures manipulating CBV: HDT and PLR, both carried out to evaluate “volume responsiveness,” i.e., whether SV and/or CO increase in response to the expansion of CBV. The evaluation was carried out in the morning for apparently normal mixed‐aged healthy men both after they had been allowed breakfast and when they had been fasting to simulate a patient scheduled for surgery. Also, there was made an evaluation of CBV by TEA and a surrogate of SV by PPI derived by photo‐plethysmography in addition to a calculation of SVV and HRV.

The SV increased during PLR while it remained stable during HDT and that was the case both after the subjects had had breakfast and when they were fastening. Also, CO tended to increase during PLR, while CO remained unchanged during HDT. Thus, HDT increased TPR with SV being similar during fastening and control. Conversely, when the subjects were exposed to PLR, TPR decreased as CO and SV increased under both control and fasted circumstances. The ~10% increase in SV by PLR was followed by an increase in TEA illustrating translocation of blood from the legs and maybe also from the splanchnic area to the thorax making healthy participants appear fluid responsive. Furthermore, HRV and SVV remained unchanged for both procedures while PPI increased only during the first minutes of PLR.

For supine healthy volunteers, the heart appears to be operating on the upper part of the cardiac function curve as SV does not increase during HDT (Bundgaard‐Nielsen et al., 2009; Harms et al., 2003; Jans et al., 2008; Van Lieshout et al., 2005) or during volume expansion (Bundgaard‐Nielsen et al., 2010a) in agreement with HDT in the present study. Similarly, Terai et al. (1996) and Gaffney et al. (1982) found that when raising the legs in supine healthy subjects SV and CO do not change. However, the PLR maneuver starts in a semi‐recumbent position, and SV increases in healthy subjects.

Jabot et al. (2009) compared the hemodynamic effects of raising the legs from the supine and semi‐recumbent position in volume responsive patients. Cardiac preload expressed as central venous pressure and right ventricular end‐diastolic area and cardiac index increased more when starting from the semi‐recumbent position than when the subjects were supine. Thus, CBV is reduced in the semi‐recumbent position as illustrated here by TEA and PLR restored CBV.

The PLR is a predictor of volume responsiveness in patients with high sensitivity and specificity for the SV response to volume expansion, (e.g., Biais et al., 2009; Dong et al., 2012; Lamia et al., 2007). However, about 70% of preoperative patients are in need of fluid (Bundgaard‐Nielsen et al., 2010b), and restoring CBV makes even normal subjects appear fluid responsive as illustrated here.

In the operating, theatre time may be important and this study evaluated the time for the hemodynamic response after 10 min for HDT and PLR. The HDT did not induce any SV and CO response. In healthy volunteers, SV and CO did not increase abruptly in response to HDT (Jennings et al., 1985) and remain unchanged for 5–15 min (Reuter et al., 2003; Van Lieshout et al., 2005). Yet, Terai et al. (1995), Terai et al. (1996) found SV and CO to increase within the first minute of HDT with the increase in SV being maintained for 10 min while CO decreased to resting values.

In patients, HDT can induce SV and CO changes within 3 (Reich et al., 1989) and 5–15 min (Gentili et al., 1988). In contrast, PLR evoked an acute increase in SV with the increase withheld throughout the 10 min period simultaneously with a decrease in TPR. This is in agreement with studies in patients showing increased SV and CO within minutes (Bertolissi et al., 2003; Boulain et al., 2002; Paelinck et al., 2003; Schrijen et al., 1991), whereas CO tends to decrease from the first minute or remain similar to baseline when exceeding 5 min evaluation at an elevated SV (Boulain et al., 2002; Schrijen et al., 1991). Thus, SV seems to be sensitive to evaluate the changes in CBV. Furthermore, the response to both HDT and PLR elicits a central hemodynamic response (i.e., translocation of blood from the lower extremities), and the HR response is controlled by sympathetic activity (Pedersen et al., 1995) but apparently not expressed by HRV in agreement with results from McHugh et al. (1994).

To evaluate fluid responsiveness “dynamic” indices are preferred over “static” predictors (e.g., central venous pressure) (Marik & Cavallazzi, 2013). Thus, the aim of this study was to evaluate whether SVV would follow the changes in CBV induced by HDT and PLR. We did not find any changes in SVV by either maneuver. The dynamic indices arise from heart‐lung interaction and therefore any change in tidal volume and/or intra‐thoracic pressure is important (Perel et al., 2013). Correlation has been found between changes in SVV and CO in relation to volume expansion in mechanically ventilated patients (Cannesson et al., 2009; Reuter et al., 2002). During spontaneous breathing, inspiration decreases intra‐thoracic pressure, whereas it increases during positive‐pressure ventilation (Wise et al., 1981). Thus, the difference between spontaneous and mechanical ventilation reflects differences in intra‐thoracic swings and, therefore, both sensitivity and specificity of SVV depend on tidal volume and pressure as a predictor of changes in CBV, SVV seems to be restricted to patients on controlled ventilation (Wiesenack et al., 2003). Although CO or SV (and thereby SVV) is the reference to detect fluid responsiveness (Michard & Teboul, 2002), not all patients are provided continuous CO and SV monitoring whereas probably most if not all patients undergoing surgery are monitored with a pulse oximeter enabling continuous monitoring of SpO_2_. The plethysmographic signal is composed of a pulsatile and a non‐pulsatile component with the pulsatile portion reflecting changes in the volume of blood in the finger during the cardiac cycle and therefore depends on SV. Thus, maneuvers that affect preload may induce proportional changes in PPI. During lower body negative pressure, PPI follows the decrease in SV in healthy spontaneous breathing subjects (McGrath et al., 2011; Van Genderen et al., 2013). Also, PPI can detect changes in CO after fluid loading in patients with septic shock (Hasanin et al., 2021; Lian et al., 2020) and during PLR in patients under intensive care (Beurton et al., 2019), but PPI as SV remained unchanged by HDT. However, PPI seems valuable for evaluating volume responsiveness in the operating theatre, as a low PPI appears associated with poor postoperative outcome (Agerskov et al., 2021) with a high correlation to other changes in systemic cardiovascular variables during general anesthesia (Højlund et al., 2020).

A strength of this study is that the participants were randomized to be fasted and control. The fasting preoperative patient may have an approximately 0.5 L volume deficit (Bundgaard‐Nielsen et al., 2010b; Jenstrup et al., 1995), and there was a decrease in HR during HDT when subjects fasted. Thus, fasting induced only moderate hypovolemia in agreement with findings by Muller et al. (2014). The present study included “mixed” healthy male subjects to simulate a population going through surgery but the results cannot be extrapolated to patients per se as they may be different if patients are on, e.g., diuretic treatment and eventual dialysis and if so when and to what extent CBV has become affected (Cai et al., 2002). Also, two participants did not complete both interventions and therefore less than the estimated sample size. We emphasize that the responses to the two procedures were consistent among the subjects but more subjects in combination with evaluations in specific patient groups or evaluation during surgery or the postoperative period would be an advantage for clinical relevance. Exposing patients in a post‐anesthesia care unit to HDT indicated those patients who had been exposed to a blood loss and indicated whether a patient is in need of fluid (Frost et al., 2017). Similar prediction has been found in the post‐anesthesia care unit exposing patients to PLR (El Hadouti et al., 2017) but the influence of the reference point used (i.e., semi‐recumbent) should be further investigated. The purpose of the present study was to evaluate PLR in a “normovolemic” population and an attempt to generalize the observation during HDT, e.g., by Harms et al. (2003). In relation to fluid responsiveness, SV and CO are often related to filling pressure (e.g., central venous and pulmonary artery wedge pressure), however, there seems to be no correlation between SV and central pressures whereas there is a relationship between SV and filling and emptying of the heart (Thys et al., 1987). Providing a fluid bolus by infusing intravenous fluids could have established whether the subjects participating in the study were not volume responsive, i.e., were normovolemic.

In summary, SV is the most sensitive variable to define whether a subject is fluid responsive as neither SVV nor PPI followed changes in SV during manipulation of CBV by tilting procedures whereas CO might be a predictor of volume responsiveness if the evaluation lasts for less than 5 min. The HDT is to be the preferred to PLR to define normovolemia as CBV is reduced in the semi‐recumbent position and raising the legs merely restores CBV and may make healthy volunteers seem fluid responsive.

## CONFLICT OF INTEREST

No conflicts of interest, financial or otherwise, are declared by the authors.

## AUTHOR CONTRIBUTION

All authors conceived and designed the research. C.S. and T.L. performed experiments. C.S. and N.H.S analyzed and interpreted the results of experiments. C.S. and N.H.S drafted the manuscript with all authors involved in the revision and approval of the final version of the manuscript.

## References

[phy215216-bib-0001] Agerskov, M. , Thusholdt, A. N. W. , Holm‐Sørensen, H. , Wiberg, S. , Meyhoff, C. S. , Højlund, J. , Secher, N. H. , & Foss, N. B. (2021). Association of the intraoperative peripheral perfusion index with postoperative morbidity and mortality in acute surgical patients: A retrospective observational multicentre cohort study. British Journal of Anaesthesia, 127, 396–404. 10.1016/j.bja.2021.06.004 34226038 PMC8451236

[phy215216-bib-0002] Bertolissi, M. , Da Broi, U. , Soldano, F. , & Bassi, F. (2003). Influence of passive leg elevation on the right ventricular function in anaesthetized coronary patients. Critical Care, 7, 164–170. 10.1186/cc1882 12720563 PMC270625

[phy215216-bib-0003] Beurton, A. , Teboul, J.‐L. , Gavelli, F. , Gonzalez, F. A. , Girotto, V. , Galarza, L. , Anguel, N. , Richard, C. , & Monnet, X. (2019). The effects of passive leg raising may be detected by the plethysmographic oxygen saturation signal in critically ill patients. Critical Care, 23, 1–10. 10.1186/s13054-019-2306-z 30658663 PMC6339274

[phy215216-bib-0004] Biais, M. , Vidil, L. , Sarrabay, P. , Cottenceau, V. , Revel, P. , & Sztark, F. (2009). Changes in stroke volume induced by passive leg raising in spontaneously breathing patients: Comparison between echocardiography and Vigileo/FloTrac device. Critical Care, 13, 1–8. 10.1186/cc8195 PMC2811910 19968880

[phy215216-bib-0005] Bogert, L. W. J. , & Van Lieshout, J. J. (2005). Non‐invasive pulsatile arterial pressure and stroke volume changes from the human finger. Experimental Physiology, 90, 437–446. 10.1113/expphysiol.2005.030262 15802289

[phy215216-bib-0006] Bogert, L. W. J. , Wesseling, K. H. , Schraa, O. , Van Lieshout, E. J. , De Mol, B. A. J. M. , Van Goudoever, J. , Westerhof, B. E. , & Van Lieshout, J. J. (2010). Pulse contour cardiac output derived from non‐invasive arterial pressure in cardiovascular disease. Anaesthesia, 65, 1119–1125. 10.1111/j.1365-2044.2010.06511.x 20860647

[phy215216-bib-0007] Boulain, T. , Achard, J.‐M. , Teboul, J.‐L. , Richard, C. , Perrotin, D. , & Ginies, G. (2002). Changes in BP induced by passive leg raising predict response to fluid loading in critically ill patients. Chest, 121, 1245–1252. 10.1378/chest.121.4.1245 11948060

[phy215216-bib-0008] Bundgaard‐Nielsen, M. , Jørgensen, C. C. , Kehlet, H. , & Secher, N. H. (2010a). Normovolemia defined according to cardiac stroke volume in healthy supine humans. Clinical Physiology and Functional Imaging, 30, 318–322. 10.1111/j.1475-097X.2010.00944.x 20545713

[phy215216-bib-0009] Bundgaard‐Nielsen, M. , Jørgensen, C. C. , Secher, N. H. , & Kehlet, H. (2010b). Functional intravascular volume deficit in patients before surgery. Acta Anaesthesiologica Scandinavica, 54, 464–469. 10.1111/j.1399-6576.2009.02175.x 20002360

[phy215216-bib-0010] Bundgaard‐nielsen, M. , Sørensen, H. , Dalsgaard, M. , Rasmussen, P. , & Secher, N. H. (2009). Relationship between stroke volume, cardiac output and filling of the heart during tilt. Acta Anaesthesiologica Scandinavica, 53, 1324–1328. 10.1111/j.1399-6576.2009.02062.x 19650800

[phy215216-bib-0011] Cai, Y. , Boesen, M. , Strømstad, M. , & Secher, N. H. (2000a). An electrical admittance based index of thoracic intracellular water during head‐up tilt in humans. European Journal of Applied Physiology, 83, 356–362. 10.1007/s004210000296 11138575

[phy215216-bib-0012] Cai, Y. , Holm, S. , Jenstrup, M. , Strømstad, M. , Eigtved, A. , Warberg, J. , Højgaard, L. , Friberg, L. , & Secher, N. H. (2000b). Electrical admittance for filling of the heart during lower body negative pressure in humans. Journal of Applied Physiology, 89, 1569–1576. 10.1152/jappl.2000.89.4.1569 11007597

[phy215216-bib-0013] Cai, Y. , Zimmerman, A. , Ladefoged, S. , & Secher, N. H. (2002). Can haemodialysis‐induced hypotension be predicted? Nephron, 92, 582–588. 10.1159/000064081 12372941

[phy215216-bib-0014] Cannesson, M. , Musard, H. , Desebbe, O. , Boucau, C. , Simon, R. , Hénaine, R. , & Lehot, J.‐J. (2009). The ability of stroke volume variations obtained with vigileo/flotrac system to monitor fluid responsiveness in mechanically ventilated patients. Anesthesia and Analgesia, 108, 513–517. 10.1213/ane.0b013e318192a36b 19151280

[phy215216-bib-0015] Cavallaro, F. , Sandroni, C. , Marano, C. , La Torre, G. , Mannocci, A. , De Waure, C. , Bello, G. , Maviglia, R. , & Antonelli, M. (2010). Diagnostic accuracy of passive leg raising for prediction of fluid responsiveness in adults: Systematic review and meta‐analysis of clinical studies. Intensive Care Medicine, 36, 1475–1483. 10.1007/s00134-010-1929-y 20502865

[phy215216-bib-0016] Cecconi, M. , De Backer, D. , Antonelli, M. , Beale, R. , Bakker, J. , Hofer, C. , Jaeschke, R. , Mebazaa, A. , Pinsky, M. R. , Teboul, J. L. , Vincent, J. L. , & Rhodes, A. (2014). Consensus on circulatory shock and hemodynamic monitoring. Task force of the European Society of Intensive Care Medicine. Intensive Care Medicine, 40, 1795–1815. 10.1007/s00134-014-3525-z 25392034 PMC4239778

[phy215216-bib-0017] Cherpanath, T. G. V. , Hirsch, A. , Geerts, B. F. , Lagrand, W. K. , Leeflang, M. M. , Schultz, M. J. , & Groeneveld, A. B. J. (2016). Predicting fluid responsiveness by passive leg raising: A systematic review and meta‐analysis of 23 clinical trials. Critical Care Medicine, 44, 981–991. 10.1097/CCM.0000000000001556 26741579

[phy215216-bib-0018] Cooke, W. H. , Ryan, K. L. , & Convertino, V. A. (2004). Lower body negative pressure as a model to study progression to acute hemorrhagic shock in humans. Journal of Applied Physiology, 96, 1249–1261. 10.1152/japplphysiol.01155.2003 15016789

[phy215216-bib-0019] Dong, Z. , Fang, Q. , Zheng, X. , & Shi, H. (2012). Passive leg raising as an indicator of fluid responsiveness in patients with severe sepsis. World Journal of Emergency Medicine, 3, 191. 10.5847/wjem.j.issn.1920-8642.2012.03.006 25215062 PMC4129784

[phy215216-bib-0020] El Hadouti, Y. , Valencia, L. , Becerra, A. , Rodríguez‐Pérez, A. , & Vincent, J. L. (2017). Echocardiography and passive leg raising in the postoperative period: A prospective observational study. European Journal of Anaesthesiology, 34, 748–754. 10.1097/EJA.0000000000000679 28719516

[phy215216-bib-0021] Frost, H. , Mortensen, C. R. , Secher, N. H. , & Nielsen, H. B. (2017). Postoperative volume balance: Does stroke volume increase in Trendelenburg’s position? Clinical Physiology and Functional Imaging, 37, 314–316. 10.1111/cpf.12306 26519213

[phy215216-bib-0022] Gaffney, F. A. , Bastian, B. C. , Thal, E. R. , & Atkins, J. M. B. C. (1982) Passive leg raising does not produce a significant sustained autotranfusion effect.pdf (190–193). 10.1097/00005373-198203000-00003 7069801

[phy215216-bib-0023] Gentili, D. R. , Benjamin, E. , Berger, S. R. , & Iberti, T. J. (1988). Cardiopulmonary effects of the head‐down tilt position in elderly postoperative patient: A prospective study. Southern Medical Journal, 81, 1258–1260. 3140385 10.1097/00007611-198810000-00014

[phy215216-bib-0024] Goldman, J. M. , Petterson, M. T. , Kopotic, R. J. , & Barker, S. J. (2000). Masimo signal extraction pulse oximetry. Journal of Clinical Monitoring and Computing, 16, 475–483. 10.1023/A:1011493521730 12580205

[phy215216-bib-0025] Harms, M. P. M. , van Lieshout, J. J. , Jenstrup, M. , Pott, F. , & Secher, N. H. (2003). Postural effects on cardiac output and mixed venous oxygen saturation in humans. Experimental Physiology, 88, 611–616. 10.1113/eph8802580 12955161

[phy215216-bib-0026] Harms, M. P. M. , Wesseling, K. H. , Pott, F. , Jenstrup, M. , Van goudoever, J. , Secher, N. H. , & Van lieshout, J. J. (1999). Continuous stroke volume monitoring by modelling flow from non‐invasive measurement of arterial pressure in humans under orthostatic stress. Clinical Science, 97, 291–301. 10.1042/CS19990061 10464054

[phy215216-bib-0027] Hasanin, A. , Karam, N. , Mukhtar, A. M. , & Habib, S. F. (2021). The ability of pulse oximetry‐derived peripheral perfusion index to detect fluid responsiveness in patients with septic shock. Journal of Anesthesia, 35, 254–261. 10.1007/s00540-021-02908-w 33616758

[phy215216-bib-0028] Højlund, J. , Agerskov, M. , Clemmesen, C. G. , Edvardsen Hvolris, L. , Foss, N. B. (2020). The peripheral perfusion index tracks systemic haemodynamics during general anaesthesia. Journal of Clinical Monitoring and Computing, 34, 1177–1184. 10.1007/s10877-019-00420-x 31705432

[phy215216-bib-0029] Jabot, J. , Teboul, J. L. , Richard, C. , & Monnet, X. (2009). Passive leg raising for predicting fluid responsiveness: Importance of the postural change. Intensive Care Medicine, 35, 85–90. 10.1007/s00134-008-1293-3 18795254

[phy215216-bib-0030] Jans, Ø. , Tollund, C. , Bundgaard‐nielsen, M. , Selmer, C. , Warberg, J. , & Secher, N. H. (2008). Goal‐directed fluid therapy: Stroke volume optimisation and cardiac dimensions in supine healthy humans. Acta Anaesthesiologica Scandinavica, 52, 536–540. 10.1111/j.1399-6576.2008.01585.x 18339159

[phy215216-bib-0031] Jennings, T. , Seaworth, J. , Howell, L. , Ssgt, L. T. , & Goodyear, C. (1985). Effect of body inversion on hemodynamics determined by two‐dimensional echocardiography. Critical Care Medicine, 13, 760–762. 10.1097/00003246-198509000-00015 4028772

[phy215216-bib-0032] Jenstrup, M. , Ejlersen, E. , Mogensen, T. , & Secher, N. H. (1995). A maximal central venous oxygen saturation (SvO2max) for the surgical patient. Acta Anaesthesiologica Scandinavica, 39, 29–32. 10.1111/j.1399-6576.1995.tb04326.x 8599293

[phy215216-bib-0033] Lamia, B. , Ochagavia, A. , Monnet, X. , Chemla, D. , Richard, C. , & Teboul, J.‐L. (2007). Echocardiographic prediction of volume responsiveness in critically ill patients with spontaneously breathing activity. Intensive Care Medicine, 33, 1125–1132. 10.1007/s00134-007-0646-7 17508199

[phy215216-bib-0034] Lian, H. , Wang, X. , Zhang, Q. , Zhang, H. , & Liu, D. (2020). Changes in perfusion can detect changes in the cardiac index in patients with septic shock. Journal of International Medical Research, 48. 10.1177/0300060520931675 PMC7418252 32776815

[phy215216-bib-0035] Marik, P. E. , & Cavallazzi, R. (2013). Does the central venous pressure predict fluid responsiveness? An updated meta‐analysis and a plea for some common sense. Critical Care Medicine, 41, 1774–1781. 10.1097/CCM.0b013e31828a25fd 23774337

[phy215216-bib-0036] Matzen, S. , Perko, G. , Groth, S. , Friedman, D. B. , & Secher, N. H. (1991). Blood volume distribution during head‐up tilt induced central hypovolaemia in man. Clinical Physiology, 11, 411–422. 10.1111/j.1475-097X.1991.tb00813.x 1934937

[phy215216-bib-0037] McGrath, S. P. , Ryan, K. L. , Wendelken, S. M. , Rickards, C. A. , & Convertino, V. A. (2011). Pulse oximeter plethysmographic waveform changes in awake, spontaneously breathing, hypovolemic volunteers. Anesthesia and Analgesia, 112, 368–374. 10.1213/ANE.0b013e3181cb3f4a 20103539

[phy215216-bib-0038] Mchugh, G. J. , Robinson, B. J. , & Galletly, D. C. (1994). Leg elevation compared with trendelenburg position: Effects on autonomic cardiac control. British Journal of Anaesthesia, 73, 836–837. 10.1093/bja/73.6.836 7880676

[phy215216-bib-0039] Michard, F. , & Teboul, J. L. (2002). Predicting fluid responsiveness in ICU patients: A critical analysis of the evidence. Chest, 121, 2000–2008. 10.1378/chest.121.6.2000 12065368

[phy215216-bib-0040] Monnet, X. , & Teboul, J. L. (2015). Passive leg raising: Five rules, not a drop of fluid! Critical Care, 19, 1–3. 10.1186/s13054-014-0708-5 25658678 PMC4293822

[phy215216-bib-0041] Muller, L. , Brière, M. , Bastide, S. , Roger, C. , Zoric, L. , Seni, G. , de La Coussaye, J.‐E. , Ripart, J. , & Lefrant, J.‐Y. (2014). Preoperative fasting does not affect haemodynamic status: A prospective, non‐inferiority, echocardiography study. British Journal of Anaesthesia, 112, 835–841. 10.1093/bja/aet478 24496782

[phy215216-bib-0042] NICE . (2013). Intravenous fluid therapy in adults in hospital. National Institute for Health and Care Excellence, 1–37. 32101393

[phy215216-bib-0043] Paelinck, B. P. , van Eck, J. W. M. , De Hert, S. G. , & Gillebert, T. C. (2003). Effects of postural changes on cardiac function in healthy subjects. European Journal of Echocardiography, 4, 196–201. 10.1016/S1525-2167(02)00167-1 12928023

[phy215216-bib-0063] Pedersen, M. , Madsen, P. , Klokker, M. , Olesen, H. L. , & Secher, N. H. (1995). Sympathetic influence on cardiovascular responses to sustained head‐up tilt in humans. Acta Physiologica Scandinavica, 155(4), 435–444. 10.1111/j.1748-1716.1995.tb09993.x 8719263

[phy215216-bib-0044] Perel, A. , Habicher, M. , & Sander, M. (2013). Bench‐to‐bedside review: Functional hemodynamics during surgery—Should it be used for all high‐risk cases? Critical Care, 17, 1–8. 10.1186/cc11448 PMC4056316 23356477

[phy215216-bib-0045] Pinsky, M. R. (2002). Functional hemodynamic monitoring. Intensive Care Medicine, 28, 386–388. 10.1007/s00134-002-1229-2 11967589

[phy215216-bib-0046] Reich, D. L. , Konstadt, S. N. , Raissi, S. , Hubbard, M. , & Thys, D. M. (1989). Trendelenburg position and passive leg raising do not significantly improve cardiopulmonary performance in the anesthetized patient with coronary artery disease. Critical Care Medicine, 17, 313–317. 10.1097/00003246-198904000-00003 2702840

[phy215216-bib-0047] Reuter, D. A. , Felbinger, T. W. , Schmidt, C. , Kilger, E. , Goedje, O. , Lamm, P. , & Goetz, A. E. (2002). Stroke volume variations for assessment of cardiac responsiveness to volume loading in mechanically ventilated patients after cardiac surgery. Intensive Care Medicine, 28, 392–398. 10.1007/s00134-002-1211-z 11967591

[phy215216-bib-0048] Reuter, D. A. , Felbinger, T. W. , Schmidt, C. , Moerstedt, K. , Kilger, E. , Lamm, P. , & Goetz, A. E. (2003). Trendelenburg positioning after cardiac surgery: Effects on intrathoracic blood volume index and cardiac performance. European Journal of Anaesthesiology, 20, 17–20. 10.1017/S0265021503000036 12553383

[phy215216-bib-0049] Rhodes, A. , Evans, L. E. , Alhazzani, W. , Levy, M. M. , Antonelli, M., Ferrer, R. , Kumar, A. , Sevransky, J. E. , Sprung, C. L. , Nunnally, M. E. , Rochwerg, B. , Rubenfeld, G. D. , Angus, D. C. , Annane, D. , Beale, R. J. , Bellinghan, G. J. , Bernard, G. R. , Chiche, J.‐D. , Coopersmith, C. , … Phillip Dellinger, R. (2017). Surviving sepsis campaign: International guidelines for management of sepsis and septic shock: 2016. Intensive Care Medicine, 43, 304–377. 10.1007/s00134-017-4683-6 28101605

[phy215216-bib-0050] Schrijen, F. , Henriquez, A. , Candina, R. , & Polu, J. (1991). Pulmonary blood volume and haemodynamic changes with legs raised in chronic lung disease patients. Cardiovascular Research, 25, 895–900. 10.1093/cvr/25.11.895 1813117

[phy215216-bib-0051] Tarvainen, M. P. , Lipponen, J. , Niskanen, J.‐P. , & Perttu Ranta‐aho, P. O. (2020). Kubios HRV software: User’s guide (version 3.4).

[phy215216-bib-0052] Tarvainen, M. P. , Niskanen, J.‐P. , Lipponen, J. A. , Ranta‐aho, P. O. , & Karjalainen, P. A. (2014). Kubios HRV—Heart rate variability analysis software. Computer Methods and Programs in Biomedicine, 113, 210–220. 10.1016/j.cmpb.2013.07.024 24054542

[phy215216-bib-0053] Terai, C. , Anada, H. , Matsushima, S. , Kawakami, M. , & Okada, Y. (1996). Effects of Trendelenburg versus passive leg raising: Autotransfusion in humans. Intensive Care Medicine, 22, 613–614. 10.1007/BF01708113 8814487

[phy215216-bib-0054] Terai, C. , Anada, H. , Matsushima, S. , Shimizu, S. , & Okada, Y. (1995). Effects of mild Trendelenburg on central hemodynamics and internal jugular vein velocity, cross‐sectional area, and flow. American Journal of Emergency Medicine, 13, 255–258. 10.1016/0735-6757(95)90194-9 7755812

[phy215216-bib-0055] Thiel, S. W. , Kollef, M. H. , & Isakow, W. (2009). Non‐invasive stroke volume measurement and passive leg raising predict volume responsiveness in medical ICU patients: An observational cohort study. Critical Care, 13, 1–9. 10.1186/cc7955 PMC2750155 19586543

[phy215216-bib-0056] Thys, D. M. , Hillel, Z. , Goldman, M. E. , Mindich, B. P. , & Kaplan, J. A. (1987). A comparison of hemodynamic indices derived by invasive monitoring and two‐dimensional echocardiography. Anesthesiology, 67, 630–634. 10.1097/00000542-198711000-00003 3499831

[phy215216-bib-0057] Truijen, J. , Westerhof, B. E. , Kim, Y.‐S. , Stok, W. J. , de Mol, B. A. , Preckel, B. , Hollmann, M. W. , & van Lieshout, J. J. (2018). The effect of haemodynamic and peripheral vascular variability on cardiac output monitoring: Thermodilution and non‐invasive pulse contour cardiac output during cardiothoracic surgery. Anaesthesia, 73, 1489–1499. 10.1111/anae.14380 30074237

[phy215216-bib-0058] van Genderen, M. E. , Bartels, S. A. , Lima, A. , Bezemer, R. , Ince, C. , Bakker, J. , & van Bommel, J. (2013). Peripheral perfusion index as an early predictor for central hypovolemia in awake healthy volunteers. Anesthesia and Analgesia, 116, 351–356. 10.1213/ANE.0b013e318274e151 23302972

[phy215216-bib-0059] van Lieshout, J. J. , Harms, M. P. M. , Pott, F. , Jenstrup, M. , & Secher, N. H. (2005). Stroke volume of the heart and thoracic fluid content during head‐up and head‐down tilt in humans. Acta Anaesthesiologica Scandinavica, 49, 1287–1292. 10.1111/j.1399-6576.2005.00841.x 16146465

[phy215216-bib-0060] Wiesenack, C. , Prasser, C. , Rödig, G. , & Keyl, C. (2003). Stroke volume variation as an indicator of fluid responsiveness using pulse contour analysis in mechanically ventilated patients. Anesthesia and Analgesia, 96, 1254–1257. 10.1213/01.ANE.0000053237.29264.01 12707116

[phy215216-bib-0061] Wise, R. A. , Robotham, J. L. , & Summer, W. R. (1981). Effects of spontaneous ventilation on the circulation. Lung, 159, 175–186. 10.1007/BF02713914 7026909

[phy215216-bib-0062] Xhyheri, B. , Manfrini, O. , Mazzolini, M. , Pizzi, C. , & Bugiardini, R. (2012). Heart rate variability today. Progress in Cardiovascular Diseases, 55, 321–331. 10.1016/j.pcad.2012.09.001 23217437

